# Determinants of Digital Health Literacy: International Cross-Sectional Study

**DOI:** 10.2196/66631

**Published:** 2025-06-30

**Authors:** Connor S Qiu, Tetiana Lunova, Geva Greenfield, Gabriele Kerr, Ömrüm Ergüven, Thomas Beaney, Benedict Hayhoe, Erik Mayer, Azeem Majeed, Ana Luisa Neves

**Affiliations:** 1 Department of Primary Care and Public Health Imperial College London London United Kingdom; 2 Institute of Global Health Innovation Imperial College London London United Kingdom; 3 iCARE Digital Collaboration Space & Secure Data Environment NIHR Imperial Biomedical Research Centre London United Kingdom; 4 Department of Surgery & Cancer Faculty of Medicine Imperial College London London United Kingdom; 5 CINTESIS@RISE Faculty of Medicine Universidade do Porto Porto Portugal

**Keywords:** digital health, digital literacy, cross-sectional, cross-sectional studies, digital health literacy, health literacy, health equity, digital determinants of health, determinants of digital health literacy, online survey, eHealth, eHealth Literacy Scale, linear regression analysis, multivariable linear regression analysis

## Abstract

**Background:**

Digital health literacy describes an individual’s ability to use digital information and tools to improve their own health. Understanding how digital health literacy varies across populations could help improve health equity. However, the determinants of digital health literacy have been scarcely evaluated.

**Objective:**

This study aims to assess the levels of digital health literacy in 4 countries (United Kingdom, Sweden, Italy, and Germany) and explore potential associations between digital health literacy and demographic characteristics and self-perceived health status.

**Methods:**

A cross-sectional online survey was disseminated to participants from the United Kingdom, Italy, Germany, and Sweden in December 2020. Digital health literacy was self-reported using the validated eHealth Literacy Scale (eHEALS; range: 0-40); low digital health literacy has been previously defined as an eHEALS score<26. Participant characteristics collected were sex, age group, ethnicity, country, and perceived overall health status. A multivariable linear regression analysis was performed to explore associations between these variables and digital health literacy.

**Results:**

A total of 6331 participants were included (51.7% female, n=3272). The mean eHEALS score was 29.2 (SD 6.8). Participant age, sex, health status, and country of residence were included in the final multivariable model. Compared to the 45- to 54-year age group, the 55 years and older age group had lower digital health literacy (β=–1.0; 95% CI –1.4 to –0.5; *P*<.001), while digital health literacy was higher in those aged 25-34 years (β=0.9; 95% CI 0.3-1.5; *P*=.002) and 35-44 years (β=0.6; 95% CI 0.1-1.2; *P*=.03). Better health status was associated with greater digital health literacy (β=0.3; 95% CI 0.2-0.4; *P*<.001). Compared to participants from Germany, those from the United Kingdom (β=2.1; 95% CI 1.7-2.5; *P*<.001) and Sweden (β=2.9; 95% CI 2.4-3.4; *P*<.001) had higher digital health literacy scores, while there was no difference with Italian participants (*P*=.399). Sex and ethnicity did not have any significant effect on digital health literacy.

**Conclusions:**

This study found significant variations in digital health literacy by age, health status, and country of residence. Targeted educational programs for vulnerable groups, particularly those of older age and poorer health status, are essential. Policies fostering accessible digital health solutions and mitigating health technology–related uncertainties for these populations are crucial for achieving optimal health outcomes.

## Introduction

Digital health literacy in the context of health care is the extent to which digital technologies are readily used by individual actors and organizations to improve population health [1,2]. It can be defined as having the knowledge and skills—or “health literacy” level—required to use digital health solutions [3]. This includes the ability to use health information from electronic sources and apply the knowledge gained to address a health problem; it is, therefore, the convergence of digital literacy and health literacy [2,4]. Higher digital health literacy is a new determinant of health and is correlated with better self-care and overall quality of life [5]. Several validated tools have been developed to quantifiably assess digital health literacy, including the commonly used eHealth Literacy Scale (eHEALS) [6,7].

How variations in digital health literacy impact health inequities is highly pertinent in this era of rapidly advancing technological progress, given the significant risk of widening disparities and structural disadvantages already present in many populations [8]. The COVID-19 pandemic accelerated digital health innovation and uptake and revealed the importance of digital health skills in accessing care and assessing health information [2,9]. This shift to increasingly digital health care provision highlighted disparities in which those better equipped to take advantage of these developments, such as those with higher educational or digital health skills and populations in higher socioeconomic categories, benefited the most [10]. As digital health literacy is essential to navigating health-related information and modern health care service environments [9], it is important to understand how digital health literacy may vary within and between populations to address health inequities.

However, digital health literacy has been understudied, and the factors that drive its variation across groups require further exploration [11]. Population and cross-country comparisons can elucidate universal best practices with respect to health policies [12] and could help inform evidence-based policy interventions needed to address any inequities in digital health skills and maximize health outcomes across populations [13]. Previous studies of populations of high- and upper-middle-income countries have reported higher digital health literacy in lower age bands and among those with higher household income, higher educational levels, and greater access to digital devices [7,14-19]. Additionally, an individual’s digital health–related skills and choices will be influenced by their experiences relating to managing their own health status [20], although it is not certain whether poorer health would limit or foster an individual’s digital health literacy skills [3]. As the popularity of digital health solutions increases, there is an imperative to further understand the most levered influences on digital health literacy levels across populations [7].

This study addresses this knowledge gap by assessing the levels of digital health literacy across 4 high-income European countries (United Kingdom, Sweden, Italy, and Germany) and exploring the relationship between digital health literacy, population demographics, and health characteristics.

By revealing factors associated with digital health literacy, this study seeks to provide health policy insights and policy recommendations to enhance digital health literacy and help address the associated digital divide in health care. Each of the selected developed countries has its commonalities and unique characteristics, allowing for some useful comparison of public policy relevant to digital health [21]. This will, in turn, add further relevant context to the formulation of valuable, generalizable, and actionable health policy to improve digital health literacy.

## Methods

### Study Design and Setting

A cross-sectional online survey was administered, which used the STROBE (Strengthening the Reporting of Observational Studies in Epidemiology) guideline for cross-sectional studies (Multimedia Appendix 1) [22].

#### Data Collection

The survey was informed by a rapid review of the literature and through expert consultation by the research team, whose feedback informed the development of the final version. This method and dataset were used in a previously published study and are available for review for further information [23]. The iterative approach used in designing this questionnaire is in keeping with accepted social research standards [24]. The question design for self-reported overall health status was informed by established health research practices [25]. Data collection took place in December 2020.

#### Study Participants and Recruitment

Participants were deemed eligible if they were 18 years or older and were able to speak, read, and write in their resident country’s language. Recruitment was carried out by YouGov, a global data and analytics group, well-established in market research. Participants were recruited by YouGov via a variety of methods, encompassing standard advertising and strategic partnerships with a broad range of websites, creating a panel of millions of people in accordance with their well-established industry standard procedures. Stratified sampling was used to recruit participants from the 4 countries of Germany, Italy, Sweden, and the United Kingdom, ensuring a nationally representative sample, in terms of age, sex, social class, and education level, for each country. This is an established methodology to collect globally representative data in health research contexts [26]. Consent to enter the study was sought from each participant only after a full explanation of the survey’s purpose had been given prior to accessing the survey on YouGov’s platform, and time allowed for consideration. All participants were free to withdraw at any time by closing the window in the browser where they were filling in the survey, and there were no consequences for withdrawal.

#### Study Variables

Digital health literacy (ie, outcome variable) was assessed by eHEALS, a validated tool that reflects an individual’s own perception of their knowledge and skills in using electronic health information [6]. This scale is a composite score of 8 items scored on a 5-point Likert scale and ranges from 0 to 40, with a score of less than 26 typically distinguishing low from high digital health literacy [27]. A histogram of the participants’ eHEALS score is shown in Multimedia Appendix 2. Predictor variables included participant characteristics (ie, sex, age, ethnicity, country, and overall health status). Participants were asked to rate their self-perceived overall health status on a scale of 1-10, with 1=very bad to 10=very good.

### Data Analysis

All participant data, including those with missing parameters, were used in the analysis. There were no missing data on sex, age, ethnicity, and country of residence. For the self-perceived overall health status, the response “Prefer not to say” or “Don’t know” was treated as missing information. Continuous data were summarized as mean (SD) and categorical data as total and relative frequencies. Linear regression was used to examine the factors associated with digital health literacy (ie, age, sex, ethnicity, country of residence, and self-perceived health scores). Unstandardized coefficients (β) and 95% CI were calculated. Characteristics with a *P* value of <.10 in univariable analyses were included in a multivariable linear regression model. Inspection of the multivariable model indicated normality in the residuals and no evidence of multicollinearity (Multimedia Appendix 3).

The significance level was set at a *P* value of <.05 for the multivariable analysis. All analyses were performed using IBM SPSS version 29.0.2.0 (20).

### Ethical Considerations

The UK Imperial College Research Ethics Committee approved and granted ethical approval for this study (reference 6847394). Participants gave their written informed consent. YouGov operates an incentive program for survey participants that is points-based. Point values are determined by survey length and are allocated upon survey completion. Respondents accumulate points for completing surveys and can redeem these for rewards, including cash and gift cards. Study data were anonymous, and data were stored on secure servers in accordance with Imperial College London's research ethics and data protection standards.

## Results

### Descriptive Characterization of Participants

This study included a total of 6331 participants, of whom 34.1% were recruited from Germany, 17.9% from Italy, 16% from Sweden, and 32% from the United Kingdom. The mean digital health literacy score (eHEALS) in this population sample was 29.2 (SD 6.8), and 28.2% (n=1787) had a score of less than 26 (“low” digital health literacy).

Over half of the respondents were White (n=3827, 60.4%), while more than one-third of the participants (n=2197, 34.7%) did not wish to disclose their ethnicity. Two-fifths (n=2684, 42.4%) of respondents were aged 55 years and older. More than half of the participants (n=4071, 64.3%) scored their overall health as 7 or more, indicating good or very good health status. The lowest scores of 3, 2, and 1 were reported by less than 7% (n=442, 6.98%) of participants. A detailed overview of the participant characteristics, including digital health literacy levels, is provided in Table 1.

**Figure 1:**
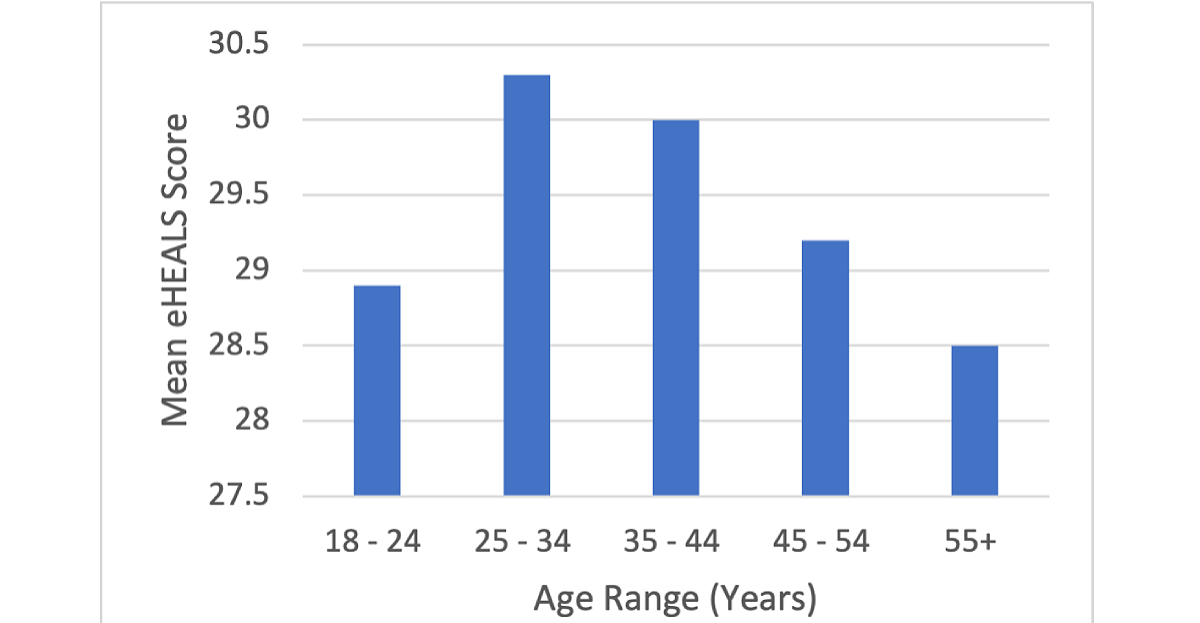
Mean eHealth Literacy Scale (eHEALS) score by age group.

**Figure 2:**
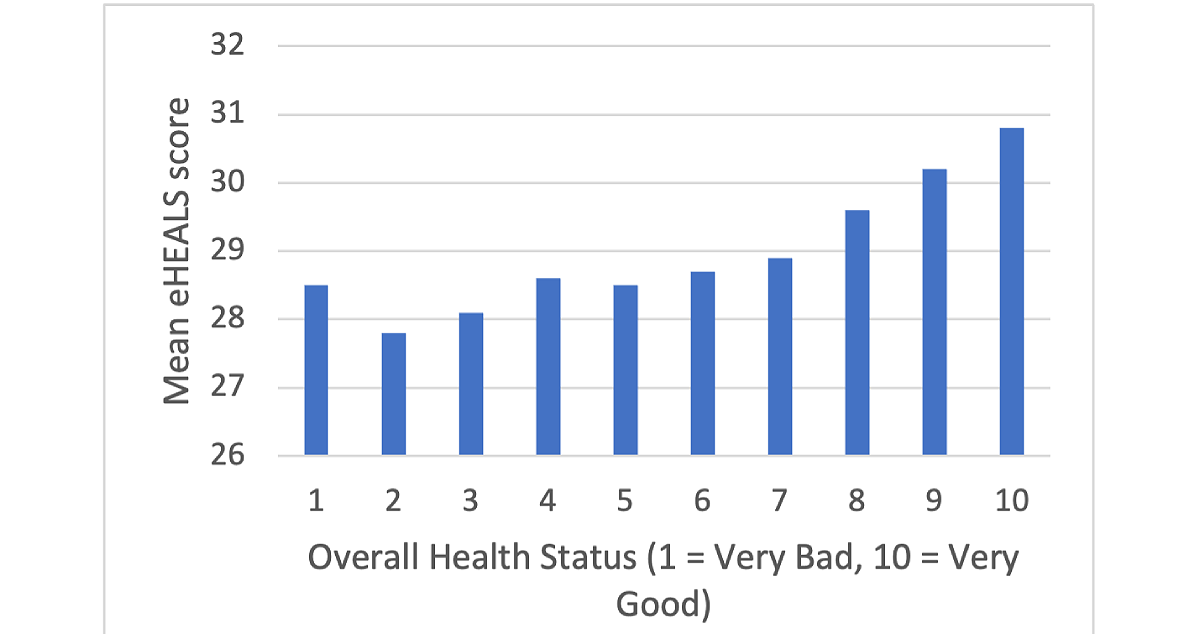
Mean eHealth Literacy Scale (eHEALS) score by participant self-reported overall health status.

Relationships between digital health literacy, age, and overall health status are shown in Figures 1 and 2, respectively.

**Table 1:** Characteristics and eHEALS^a^ score of the survey participants (N=6331).

Characteristics		Participants, n (%)	eHEALS score, mean (SD)
**Sex**			
	Female	3272 (51.7)	29.3 (6.6)
	Male	3059 (48.3)	29.0 (7.0)
	Missing	0 (0)	0
**Age (years)**			
	18-24	505 (8)	28.9 (6.2)
	25-34	998 (15.8)	30.3 (6.5)
	35-44	1001 (15.8)	30.0 (6.2)
	45-54	1140 (18)	29.2 (6.7)
	55 and older	2687 (42.4)	28.5 (7.1)
	Missing	0 (0)	0
**Ethnicity**			
	Asian	77 (1.2)	30.2 (6.0)
	Black, African, or Caribbean	27 (0.4)	28.0 (5.0)
	White	3827 (60.4)	29.8 (6.6)
	Mixed and multiple ethnic groups	150 (2.4)	29.4 (6.5)
	Other	51 (0.8)	28.4 (8.6)
	Prefer not to say (GDPR^b^)	2199 (34.7)	28.0 (7.0)
	Missing	0 (0)	0
**Country**			
	Germany	2161 (34.1)	28.0 (7.0)
	Italy	1131 (17.9)	28.0 (6.5)
	Sweden	1015 (16)	31.0 (6.7)
	United Kingdom	2024 (32)	30.2 (6.4)
	Missing	0 (0)	0
**Perceived overall health status**			
	1=very bad	76 (1.2)	28.5 (9.2)
	2	85 (1.3)	27.8 (7.3)
	3	281 (4.4)	28.1 (7.1)
	4	371 (5.9)	28.6 (7.0)
	5	606 (9.6)	28.5 (6.6)
	6	623 (9.8)	28.7 (6.3)
	7	1169 (18.5)	28.9 (6.5)
	8	1425 (22.5)	29.6 (6.4)
	9	818 (12.9)	30.2 (6.6)
	10=very good	661 (10.4)	30.8 (7.4)
	“Don’t know”	75 (1.2)	25.3 (7.0)
	“Prefer not to say”	94 (1.5)	25.8 (7.3)
	Prefer not to say (GDPR)	47 (0.7)	26.6 (7.2)
	Missing	0 (0)	0

^a^eHEALS: eHealth Literacy Scale.

^b^GDPR: General Data Protection Regulation

### Factors Associated With Digital Health Literacy

In the univariable analysis, male sex, the United Kingdom and Swedish residency, higher health status, and being aged 25-34 or 35-44 years were associated with higher digital health literacy, relative to their respective reference categories (Table 2). Meanwhile, being aged 18-24 years and 55 years and older were associated with lower digital health literacy levels in the univariable analysis relative to the 45-54 years age group. No statistically significant association between ethnicity and digital health literacy score was detected.

All variables that were statistically significant in the univariable analysis were included in the multivariable model.

In the multivariable model, sex was no longer associated with digital health literacy after adjustment for age, country of residence, health status, and age in the multivariable model, (Table 2). Country, age, and perceived health status remained significant predictors of health literacy. Relative to participants from Germany, participants from the United Kingdom (β=2.1; 95% CI 1.7-2.5; *P*<.001) and Sweden (β=2.9; 95% CI 2.4-3.4; *P*<.001) had higher digital health literacy scores. No evidence for a difference in digital health literacy scores was found between German and Italian participants (*P*=.40). Participants aged 55 years and older had lower digital health literacy on average (β=–1.0; 95% CI –1.4 to –0.5; *P*<.001), compared to participants aged 44-55 years. Meanwhile, higher digital health literacy was found in those aged 25-34 years (β=0.9; 95% CI 0.3-1.5; *P*=.002) and 35-44 years (β=0.6; 95% CI 0.1-1.2; *P*=.03). Participants aged 18-24 years had lower digital health literacy scores on average than those aged 44-55 years; however, this difference was not statistically significant (β=–0.3; 95% CI –1.0 to 0.5; *P*=.49). Digital health literacy showed a positive correlation with self-perceived health score (β=–0.3; 95% CI 0.2-0.4; *P*<.001).

**Table 2:** Coefficients (β) and associated 95% CI from univariable and multivariablea linear regression models of 6331 participants’ digital health literacy scores.b

Characteristics		Univariable analysis			Multivariable analysis^a^	
		β (95% CI)	*P* value	β (95% CI)		*P* value
**Sex**						
	Female	0.285 (–0.050 to 0.620)	.10	0.311 (–0.018 to 0.639)		.06
	Male	[Ref]^b^	[Ref]	[Ref]		[Ref]
**Age (years)**						
	18-24	–0.331 (–1.038 to 0.377)	.36	–0.251 (–0.964 to 0.461)		.49
	25-34	1.141 (0.567-1.714)	<.001^c^	0.916 (0.345-1.486)		.002^d^
	35-44	0.842 (0.268 -1.415)	.004^d^	0.621 (0.056-1.186)		.03^e^
	45-54	[Ref]	[Ref]	[Ref]		[Ref]
	55 and older	–0.706 (–1.174 to –0.238)	.003^d^	–0.983 (–1.445 to –0.521)		<.001^c^
**Country**						
	Germany	[Ref]	[Ref]	[Ref]		[Ref]
	Italy	–0.041 (–0.520 to 0.439)	.87	–0.207 (–0.689 to 0.274)		.40
	Sweden	2.979 (2.482-3.477)	<.001^c^	2.923 (2.423-3.422)		<.001^c^
	United Kingdom	2.222 (1.818-2.627)	<.001^c^	2.105 (1.699-2.511)		<.001^c^
Health score		0.351 (0.270 - 0.431)	<.001^c^	0.312 (0.233-0.392)		<.001^c^
**Ethnicity**						
	Asian	0.364 (–1.123 to 1.851)	.63	—^f^		—
	Black	–1.857 (–4.352 to 0.639)	.14	—		—
	White	[Ref]	[Ref]	—		—
	Mixed	–0.430 (–1.505 to 0.645)	.43	—		—
	Other	–1.425 (–3.246 to 0.396)	.12	—		—

^a^The multivariable model included age, sex, country, and health score as predictor variables.

^b^Reference levels of categorical predictors are denoted by “[Ref].”

^c^*P*≤.001.

^d^*P*≤.01.

^e^*P*≤.05.

^f^Not applicable.

## Discussion

### Principal Results

The average digital health literacy score (eHEALS) in the survey population was 29.2 (SD 6.8; Multimedia Appendix 2), which would be defined as “high literacy” (eHEALS>26) by the eHEALS scale [6]. The 25-34 and 35-44 age groups had the highest digital health literacy levels, and the 55 years and older age group had the lowest. (Table 2). Participants from the United Kingdom and Sweden had higher overall digital health literacy levels than German participants, after adjusting for age and sex. Higher self-reported overall health status was associated with higher digital health literacy levels. No evidence for a relationship was found between digital health literacy and either sex or ethnic group, in unadjusted or adjusted analyses.

### Comparison With Prior Work

#### Background

The overall mean digital health literacy score in this sample was high and varied by age and country of residence. Similar findings have been reported in several other observational studies, which report the United Kingdom and Swedish populations as having relatively high digital health literacy, in contrast to studies of residents of Germany or Italy [28-31]. A sizable minority of participants (n=1785, 28.2%) had a “low” health literacy score, which indicates a reduced ability to access and use digital health services effectively and is associated with worse patient engagement and health improvement [5,32]. The simple histogram of eHEALS (Multimedia Appendix 2) shows a left-skewed and multimodal distribution, there are peaks at 8, 16, 24, 32, and 40 which suggests that individual participants were often more likely to select the same rating in the Likert scale across all eHEALS question domains, for example, “agree” for all the questions. This is mirrored in other work and would be supportive of the validity of eHEALS and its design as a single-factor model [33]. Previous work suggests that normal distributions with a left-skew are within expectation for this kind of study [34].

#### Age

Older adults in this survey had lower digital health literacy. This is in line with a recent meta-analysis showing that digital health literacy decreases with increasing age [7]. Older people also engage less with digital health solutions than younger adults [32,35]. This trend applies to digital health literacy in the broadest sense and as a core skill and is also found in other countries, such as the United States and China [15,36]. In Sweden, older people have a subjective sense that it is difficult to take advantage of digital health solutions [37], and literature also supports that the younger age groups are more able to take advantage of digital health tools [38]. In line with this, some European countries, such as Hungary, consider that digital health literacy education would be well targeted at the older generations [39]. However, the literature can be mixed on this topic. In the 40-64 age group in South Korea, digital health literacy increased with age [40]. Additionally, when provided with training, older adults are able to take advantage of digital solutions for the benefit of their health [41].

#### Country

Cross-country variations in digital health literacy were observed in this study, with the United Kingdom and Sweden having higher scores compared to Germany and Italy. These findings are consistent with a previous analysis of European Commission data [42]. The reason behind these differences is multifactorial, occurring through educational policies, cultural attitudes, and economic factors unique to each country. For instance, the United Kingdom’s 2017 public health digital strategy and 2016 child health information strategy, and Sweden’s “Vision e-hälsa 2025” (implemented since 2017), focus on improving digital health skills in the population and promoting digital literacy through partnerships with the private sector and through funding for digital infrastructure [43-45]. In contrast, digital policies in Germany focused more on industrial digitalization rather than digital health skills in the general population [19,29]. At the same time, Germany and Italy have started implementing comprehensive digital skills policies more recently and have been reported to face a significant digital divide in society [19,29,31].

#### Self-Reported Health Status

Our study found a positive association between higher digital health literacy and self-reported health status, independent of age. This finding is supported by previously published literature: a study in Germany has found digital health literacy to be weakly correlated with higher overall health status [46] systematic review described that higher digital health literacy has been associated with better behavioral, cognitive, and some physical health outcomes in older adults [47]. Greater digital health literacy may enable individuals to better self-manage their own health, thus improving their health status. Conversely, poor health may physically or mentally limit an individual’s ability to engage with digital health resources, and therefore their ability to exercise digital health skills. There may also be a confounding third factor not considered in this analysis, such as educational level, where those with less education are more likely to have both poorer health and lower digital health literacy [7,47].

#### Sex and Ethnicity

This study found no difference in digital health literacy by sex. This finding is consistent with several previous studies [7,15-18]. While there may be differences in health outcomes by sex, these are often diminished after accounting for other factors, particularly education and socioeconomic status [7,16]. Given educational attainment is similar between sexes in the populations studied [48], it is therefore understandable that no difference in digital health literacy by gender in this study. Similarly, no association between ethnicity and digital health literacy was detected, consistent with some previous research [7,49]. However, the proportion of participants who chose not to disclose their ethnicity was high in this study (n=2197, 34.7%), which may have inhibited the ability to statistically detect differences in digital health literacy between ethnic groups. Additionally, the role of ethnicity in experiences and outcomes relating to health care can vary greatly depending on the cultural context. Depending on the population under examination, factors such as age, educational level, and language may vary by ethnicity and contribute to differences observed in digital health literacy between ethnic groups [49,50]. Given that populations from multiple countries were surveyed, this analysis assumes a homogeneous effect of both sex and ethnicity across multiple cultural contexts. Analyses of subpopulations from these countries, with sufficient power and incorporating a wider range of potential contributing factors, may uncover sex or ethnic differences in digital health literacy. Further analyses with more comprehensive datasets should also explore uncovered interactions between variables of interest. Additionally, although there was no observed difference in digital health literacy by sex or ethnicity, differences may nevertheless persist in the form of varied types of use and experiences with digital health tools [9,18,51].

### Strengths and Limitations

Key strengths of this study include the use of stratified sampling to recruit large and nationally representative samples, and the use of eHEALS, a validated objective measure of participant digital health literacy. Digital health literacy, to the best of our knowledge, has not been studied in nationally representative samples of these 4 countries, nor has it been compared in this population-wide context.

The findings of this study must be interpreted in light of its limitations. First, the generalizability of the study’s findings is restricted to countries with similarly high levels of gross domestic product and health expenditure as the 4 countries studied here [52]. Future research into patterns and determinants of digital health literacy in countries with lower overall income and health expenditure would be valuable.

Second, the directionality of the associations cannot be determined due to the cross-sectional nature of the survey. Consequently, any causal relationships between age or health status and digital health literacy cannot be established. It is not possible to understand whether an individual’s poorer health resulted in lower digital health literacy, or vice versa. Therefore, longitudinal or experimental studies are needed to explore the causal relationship between the various factors and digital health literacy. Furthermore, health status was self-reported at the time of the survey; therefore, this variable may subjectively describe the health situation of the participant over the past year, rather than the longer-term health status of the surveyed individual.

Third, the recruitment method used may have self-selected participants more familiar with online tools, as the survey was disseminated online. There may therefore be an underrepresentation of participants lacking the ability to access online tools in the survey sample.

Fourth, several key factors known to be associated with (digital) health literacy were not captured in this survey. Educational level, digital literacy, and household income are major determinants of digital health literacy [7,14,49]. This, however, was deemed beyond the scope of the survey, which was originally designed to assess the use and impact of virtual primary care on quality and safety [53]. Additionally, although ethnicity was surveyed, a high proportion of participants (n=2197, 34.7%) did not report ethnicity, so there was insufficient power to investigate interactions between ethnicity and other predictors. This limits the power of the finding that ethnicity is not implicated in digital health literacy levels.

Finally, as eHEALS was developed in 2006 [6], the tool may not capture the full extent of digital health literacy in the modern day, where health technology is advancing at pace and there is widespread use of social media. Consideration of other metrics, such as the Digital Health Literacy Instrument, is an area for future work [54]. The eHEALS is further limited by its factor structure, where not all question items measure solely digital health literacy [55]. Nevertheless, eHEALS remains the predominant tool used to measure digital health literacy [56]. There is potential value in analyzing eHEALS questions separately or in discrete groupings of 2 to 3 questions if eHEALS is considered to be, in actuality, a multidimensional tool; however, this is beyond the scope of this study.

### Implications for Policy and Practice

Previous research to measure average digital health literacy across Europe found that it seems strongly correlated with overall health literacy levels [57]. Therefore, it would be prudent for countries to consider creating synergistic health literacy and digital health literacy educational programs tailored differently toward younger adult and older adult age ranges. The digital health educational needs of these age groups are very different, so countries should consider targeted educational health policies that adapt to the evolving digital and health literacy levels of the population over time. A gap in this work is understanding the effect of socioeconomic status and vulnerable population status on digital health literacy, and this needs to be considered and addressed [58].

Digital health solutions should consider the relative levels of digital health literacy across countries and tailor their product offering to their target market, ideally developing their solutions based on user-centered research that maximizes usability. Digital health reimbursement strategies will play an important part in the speed of equitable development of such solutions, and therefore, countries need to understand the digital health literacy levels of their countries to best adapt or introduce helpful digital health reimbursement models [59]. Due to fragmentation and possible cross-country migration and competition between services, cross-continent collaboration, and health policy that spans countries are key to increasing the adoption and availability of digital health solutions and improving digital health literacy levels [60]. This is an area where further research is needed to generate a more robust evidence base [61].

There is also inherent uncertainty in the adoption and development of new technology, such as generative artificial intelligence (AI) and spatial computing [62]. These technological advancements pose new challenges for countries in maintaining digital health literacy levels and exacerbate the digital divide [63]. It is now critical to generate an in-depth understanding of the needs of the population from a digital health literacy standpoint by segmenting the population's needs, updating this information periodically, and strategically developing tailored support to those needs. Since older people were found to have lower digital health literacy, it is crucial to develop targeted interventions to bridge this gap. Investing in educational initiatives, such as in-person workshops, tutorials, or user-friendly training programs, can empower older service users to navigate digital technologies more effectively [64]. Special needs of older people can also be met by simplifying the interfaces of digital tools and apps, tailoring the size of the display icons and volume settings, and incorporating voice-assisted technologies [65].

As shown in other fields, advances in AI, particularly generative AI, may improve productivity by 34% in novice and low-skilled workers [66]. As such, countries need to understand the effects of new technology on health access and prepare their populations, and indeed health care professionals [67], to access such technology equitably to empower efficient and cost-effective self-care in aging populations in response to finite resources and increasing health spend [68].

Previous studies have highlighted the importance of digital health literacy in improving population health outcomes and reducing health inequalities [69]. This is likely grounded in the ability of those with high digital health literacy levels to make use of digital health information in a way that improves their health through awareness and comfort with technology in the digital domain [70].

### Conclusions

As health systems become ever-more digitized, promoting digital health literacy is essential for improved health outcomes. This study reinforces that there are significant variations in digital health literacy by age, health status, and country of residence. Participants aged 55 years and older had lower digital health literacy relative to younger age groups. Better health status was associated with greater digital health literacy, whereas sex and ethnicity did not have any significant effect on digital health literacy. To address these disparities, it is imperative to implement targeted educational programs that cater to the specific needs of vulnerable and older populations. Policy makers should promote accessible digital health solutions to meet the needs of diverse populations and encourage their adoption among vulnerable populations. By identifying best practices and addressing common challenges, policy makers can leverage the potential of digital health to enhance health care access and quality.

## Data Availability

Any additional data are available upon reasonable request from the corresponding author.

## Appendix

Multimedia Appendix 1 STROBE (Strengthening the Reporting of Observational Studies in Epidemiology) checklist.

Multimedia Appendix 2 Simple histogram of eHEALS (eHealth Literacy Scale) scores distribution.

Multimedia Appendix 3 Simple histogram of standardised residuals.

## Abbreviations

AI: artificial intelligence
eHEALS: eHealth Literacy Scale
STROBE: Strengthening the Reporting of Observational Studies in Epidemiology
