# Supplementary figures and images for: Determinants of Digital Health Literacy: International Cross-Sectional Study
Source: J Med Internet Res. 2025 Jun 30;27:e66631. doi: 10.2196/66631 (PMC12260470; doi:10.2196/66631)

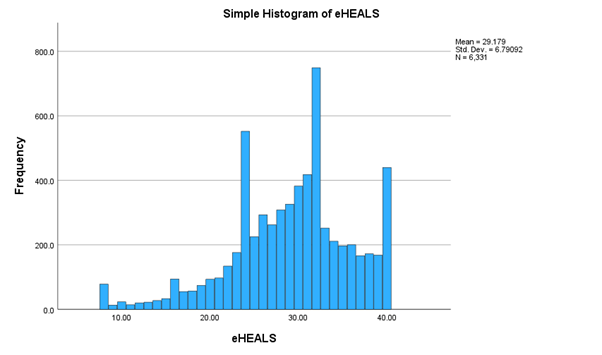

Supplement: Multimedia Appendix 2 [file jmir_v27i1e66631_app2.png]

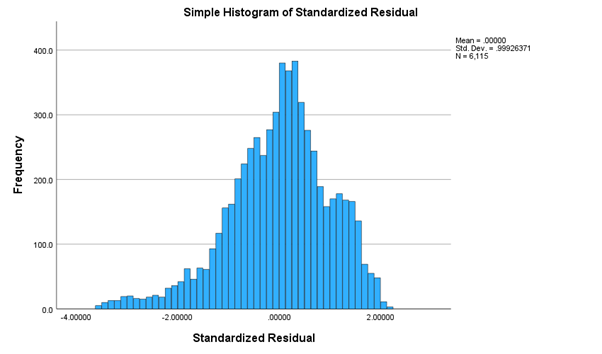

Supplement: Multimedia Appendix 3 [file jmir_v27i1e66631_app3.png]
